# Fraction I of Whey Protein Hydrolysates Improves Lipid Stability and Water Retention in Ground Pork During Freeze–Thaw Cycles

**DOI:** 10.3390/foods14234161

**Published:** 2025-12-04

**Authors:** Yifan Yin, Yina Yin, Jia Li, Haoyu Lv, Xuefeng Tang, Chao Wang, Qingtian Dong

**Affiliations:** College of Life Sciences, Yantai University, Yantai 264005, China

**Keywords:** WPH, antioxidant capacity, ground pork, freeze and thaw cycles, water retention capacity

## Abstract

This study investigated the potential of Fraction I (FI, <1 kDa), a peptide fraction derived from whey protein hydrolysates (WPH) exhibiting strong in vitro scavenging activity against DPPH, superoxide, and hydroxyl radicals, to mitigate quality deterioration in ground pork subjected to up to seven freeze–thaw (F–T) cycles. Ground pork samples were supplemented with FI at three concentrations (5%, 10%, and 15%) and analyzed for oxidative and physicochemical changes. Successive F–T cycles markedly promoted lipid oxidation, as indicated by increased peroxide value and thiobarbituric acid reactive substances (TBARS), and overall quality deterioration, evidenced by a rise in acid value and pH along with a decrease in G″. Incorporation of 10% FI effectively inhibited these oxidative reactions and improved the water-holding capacity of ground pork. These results demonstrate that among the tested concentrations, FI at 10% most effectively enhances oxidative stability and maintains water distribution, thereby preserving the quality of ground pork during multiple F–T cycles.

## 1. Introduction

The storage stability of meat is largely determined by its sensory appearance, textural firmness, color, flavor, and nutritional composition, all of which can be altered during freezing and thawing processes [1]. Freezing remains the most practical approach for maintaining meat freshness over extended periods [2]. However, temperature fluctuations during storage, transportation, or retail display often result in multiple freeze–thaw (F–T) cycles, which inevitably induce physical and biochemical deterioration in muscle tissue. This deterioration is manifested as reduced water retention, texture degradation, and enhanced lipid and protein oxidation [3].

The underlying mechanisms are primarily associated with the formation and recrystallization of ice crystals, which rupture cell membranes and aggravate mechanical damage [4]. The cavities formed after ice melting facilitate undesirable biochemical reactions, including protein denaturation and lipid oxidation, further accelerating meat quality loss [5]. Of particular concern is lipid oxidation, an inevitable event during multiple F–T cycles [6]. This process is driven by the release of redox-active molecules from damaged muscle microstructure, leading to the formation of primary and secondary oxidation products responsible for rancid flavors and overall quality impairment [7,8]. Moreover, With each successive F–T cycle, muscle fiber integrity weakens, leading to noticeable changes in surface color, texture softening, flavor decline, and moisture exudation, ultimately diminishing the sensory and commercial value of meat products [9,10].

To mitigate these adverse effects, antioxidant ingredients are frequently incorporated into meat formulations. In recent years, growing attention has been paid to natural antioxidant peptides obtained from food proteins due to potential safety issues of synthetic antioxidants [11]. Among available protein sources, whey protein hydrolysate (WPH)—obtained from the enzymatic treatment of native whey protein, a cheese by-product—has been recognized as a functional ingredient. The hydrolysis process breaks specific peptide bonds, yielding peptides and free amino acids with strong antioxidant potential [12]. Numerous studies have reported that protein hydrolysates can bind to muscle proteins through molecular interactions, effectively retarding oxidative degradation [13,14].

Our prior experiments revealed that WPH effectively retards oxidative degradation in frozen–thawed pork [15]. However, the individual peptide fractions contributing to this protection and their specific mechanisms of action remain poorly understood. We hypothesized that the antioxidant efficacy of WPH is primarily concentrated in its low-molecular-weight fraction (<1 kDa), and that this fraction would most effectively mitigate freeze–thaw-induced deterioration in ground pork by concurrently inhibiting lipid oxidation and improving water retention.

The novelty of this research lies in the systematic identification of the most active peptide fraction from WPH and a comprehensive evaluation of its dual functionality (antioxidant and water-retaining) in a real meat system during repeated F–T cycles, which has not been previously elucidated. Therefore, the present study aimed to (1) separate WPH by molecular weight to identify the most antioxidant-active fraction; (2) assess its efficacy in controlling lipid oxidation and moisture retention in ground pork during successive F–T cycles; and (3) determine the optimal application level for quality preservation.

## 2. Materials and Methods

### 2.1. Chemicals and Materials

Whey protein isolate (95% purity) and native whey protein powders were purchased from Davisco Foods International (Le Sueur, MN, USA). Fresh longissimus dorsi muscle and back fat were sourced from a local meat processing plant in Yantai, Shandong Province, China. The enzyme Alcalase 2.4 L (6 × 10^4^ U g^−1^) was supplied by Novo Nordisk Biochem (Franklinton, NC, USA). Reagents including phosphate buffer, bromophenol blue (BPB), 5,5′-dithiobis(2-nitrobenzoic acid) (DTNB), ethylenediaminetetraacetic acid (EDTA), and butylated hydroxyanisole (BHA) were obtained from Sigma-Aldrich (St. Louis, MO, USA). All other chemicals used in this study were of analytical grade and purchased from Sinopharm Chemical Reagent Co. (Shanghai, China).

### 2.2. Preparation of Whey Protein Hydrolysate

Whey protein hydrolysate (WPH) was produced following a modified protocol adapted from Peng et al. [16]. Native whey protein (NWP) was first dissolved in deionized water to obtain a 10% (*w*/*v*) solution and preheated at 95 °C for 5 min to thermally denature the proteins and inactivate potential endogenous enzyme activity. The pH of the mixture was then adjusted and maintained at 8.5 using 1 mol/L NaOH. Hydrolysis was carried out by adding alkaline protease at an enzyme-to-substrate ratio of 2:100 (*w*/*w*), and the reaction mixture was incubated at 65 °C for 8 h in a thermostatic water bath. After enzymolysis, the pH typically decreased due to the release of amino and carboxyl groups during peptide bond cleavage, and enzyme deactivation was achieved by heating the solution in boiling water for 10 min.

### 2.3. Determination of DPPH Free Radical Scavenging Capacity

The DPPH free radical scavenging capacity of the hydrolysate was evaluated over an 8-hour timeframe using a slightly modified method based on Shen [17]. Initially, 100 µL of the hydrolysate solution was mixed with 50 µg/mL DPPH solution and thoroughly blended for 1 min. Subsequently, the mixtures were incubated in darkness for 30 min. After incubation, the absorbance of the samples was determined at 517 nm with a UV-Vis spectrophotometer (SHIMADZU UV-2450, Tokyo, Japan). The DPPH radical scavenging activity of the samples was calculated using the following formula:

$$\mathrm{DPPH}\;\mathrm{radical}=\mathrm{scavenging}\;\mathrm{activity}=\frac{A0\;-\;(A1\;-\;A2)}{A0}\;\times \;100\%$$

where A_0_ is the DPPH absorbance, A_1_ is the sample and DPPH mixture absorbance, and A_2_ is the absorbance of the sample.

### 2.4. Determination of Ferric Reducing Antioxidant Power

The hydrolysate produced after 4 h of enzymatic treatment was fractionated via ultrafiltration membranes (MWCO: 1 and 3 kDa, Millipore, Bedford, MA, USA) to separate peptides into three molecular weight fractions: Fraction I (FI, <1 kDa), Fraction II (FII, 1–3 kDa), and Fraction III (FIII, >3 kDa). The ferric reducing antioxidant power (FRAP) of these fractions was determined via a modified method adapted from Chen et al. [18], with 1.0 mmol/L FeSO_4_ used as the calibration standard. For the preparation of FRAP working solution, 2.5 mL of 10 mmol/L TPTZ solution, 25 mL of 0.3 mol/L acetate buffer (pH 3.6), and 2.5 mL of 20 mmol/L ferric chloride solution were sequentially mixed in deionized water. Subsequently, 2.4 mL of the freshly prepared reagent was combined with 0.1 mL of sample solution (10 mg/mL, dry weight basis). The reaction mixtures were incubated at 37 °C for 10 min in the dark to avoid light-induced degradation. Absorbance was then measured in duplicate at 593 nm using a UV–Vis spectrophotometer (Cary 60, Agilent Technologies, Santa Clara, CA, USA). The FRAP value of each sample was calculated from a standard curve established with FeSO_4_·7H_2_O solutions at concentrations ranging from 100 to 1000 μmol/L.

### 2.5. Determination of Free Radical Scavenging Capacity by Electron Spin Resonance

The free radical scavenging ability of whey protein hydrolysate (WPH) fractions was determined using an electron spin resonance (ESR) spectrometer, following a modified method based on Peng et al. [16]. Three radical species were analyzed, including 1,1-diphenyl-2-picrylhydrazyl (DPPH•), hydroxyl (•OH), and superoxide anion (O_2_•^−^). The intensity of the DPPH• signal was quantified by recording the amplitude of the third spectral peak, while the response of the hydroxyl radical was represented by the height of the second peak. For the superoxide anion, the first peak in the ESR spectrum was used for signal quantification. The scavenging efficiency for each radical type was calculated according to the following formula:

$$Scavenging\;rate\;\left(\%\right)\;=\left[\frac{H_{0}-H}{H_{0}}\right]\times 100\%$$

where H_0_ and H represent the ESR signal intensities of the control (blank) and the sample, respectively.

### 2.6. Preparation of Ground Pork

Fresh longissimus dorsi muscles and back fat were obtained from 3 healthy crossbred pigs (Duroc × Landrace × Yorkshire, 6-month-old, body weight 90–100 kg) at a local meat processing plant in Yantai, Shandong Province, China. Muscles from each pig were processed independently as separate biological replicates. The cleaned muscle tissue and pre-weighed back fat were then minced using a grinder (Midea MJ-LZ25Easy225, Foshan, China) equipped with a 4-mm plate and blended at a lean-to-fat ratio of 7:3 to obtain uniform ground pork. The mixture was randomly divided into six 500 g portions: one served as the untreated control, while the remaining five were supplemented with different additives:10% natural whey protein (NWP), 5% fraction I (FI), 10% FI, 15% FI, and 0.02% butylated hydroxyanisole (BHA) as the positive control. Each sample was then homogenized with 1.5% (*w*/*w*) sodium chloride (NaCl) solution to ensure even distribution of additives. Subsequently, 75 g of each treatment was packed individually into polyethylene bags. All packaged samples were frozen at −18 °C for 5 days and then thawed at 4 °C for 12 h until the internal temperature reached 0–2 °C, simulating one freeze–thaw (F–T) cycle. This freezing and thawing sequence was repeated for 3, 5, and 7 cycles following the described protocol.

### 2.7. Determination of Acid Value

The acid value of ground pork was determined following the method of Liu and Tang [19] with minor adjustments. Briefly, 5.0 g of minced meat was accurately weighed and transferred into a conical flask, to which 30 mL of a petroleum ether–ethanol solution (1:1, *v*/*v*) was added. The mixture was gently swirled to extract the lipid fraction and then titrated directly with 0.05 mol/L ethanolic potassium hydroxide until a persistent pale pink endpoint appeared, remaining stable for approximately 30 s in the presence of phenolphthalein indicator. The acid value, expressed as milligrams of KOH required to neutralize the free fatty acids in one gram of sample lipid, was calculated according to the consumed volume of titrant. The specific analytical steps for determining the acid value were conducted as follows:

$$\mathrm{Acid}\;\mathrm{value}\;=\;\frac{V\;\times \;c\;\times \;56.11}{m}$$

where V is the standard volumetric potassium hydroxide solution’s employed volume in milliliters; c is the concentration in moles per liter of the standard volumetric potassium hydroxide solution employed; and m is the mass in grams of the test sample of samples.

### 2.8. Determination of Peroxide Value

The peroxide value (POV) of ground pork was determined via a modified method based on Wang et al. [20]. Briefly, 5.0 g of ground meat was homogenized for 1 min in 20 mL of a chloroform–methanol mixture (1:1, *v*/*v*). Thereafter, 6.16 mL of 0.5% sodium chloride solution was added, and the mixture was vortexed for 30 s to promote phase separation prior to centrifugation at 1800× *g* for 6 min at 4 °C. A 5 mL aliquot of the lower organic phase was carefully extracted with a glass syringe and transferred to a clean glass tube. To this extract, 3 mL of chloroform–methanol (1:1), 100 µL of 3.94 M ammonium thiocyanate, and 100 µL of 18 mM ferrous chloride were added sequentially, followed by brief vortexing for 10 s. The resulting solution was incubated at 25 °C for 20 min, after which its absorbance was measured at 500 nm using a UV-1601 spectrophotometer (Shimadzu, Tokyo, Japan).

### 2.9. Determination of Thiobarbituric Acid-Reactive Substances

The thiobarbituric acid reactive substances (TBARS) content in ground pork was determined via the method described by Gan [21] with minor adjustments. Briefly, 5 g of ground sample was homogenized with 20 mL of ultrapure water for 10 s. Subsequently, 50 mL of 7.5% trichloroacetic acid (TCA) solution supplemented with 1 g of EDTA was added, and the mixture was gently stirred at 50 °C for 30 min. The homogenate was subjected to filtration, and 5 mL of the resulting filtrate was mixed with an equal volume of 2-thiobarbituric acid solution. This reaction mixture was incubated in a 90 °C water bath for 30 min and then cooled to room temperature. The absorbance of the final solution was measured at 532 nm. The TBARS values were calculated based on a standard curve prepared using 1,1,3,3-tetraethoxypropane (TEP) or malondialdehyde (MDA) standard solutions and expressed as malondialdehyde (MDA) equivalents in milligrams per kilogram of pork tissue.

### 2.10. Determination of pH

The pH of ground pork was measured following the method reported by Hu et al. [22] with modest revisions. Briefly, 10 g of ground sample was mixed with 90 mL of distilled water and fully homogenized using a laboratory-grade homogenizer. The homogenate was subsequently centrifuged at 2500 rpm for 5 min to obtain a clear supernatant. The pH electrode was directly immersed in the supernatant for the determination of hydrogen ion concentration. Each analysis was performed in triplicate, and the average of the three measurements was taken as the final pH value.

### 2.11. Dynamic Rheological Properties

A 5.0 g portion of ground pork was positioned between the parallel plates (diameter: 40 mm) of a rheometer (Discovery Hybrid Rheometer, TA Instruments, New Castle, DE, USA), maintaining a 1.5 mm gap. The sample was heated from 20 °C to 80 °C at a constant rate of 1 °C per minute at a fixed frequency of 1 Hz and a strain of 0.5% (within the linear viscoelastic region) to evaluate its dynamic rheological behavior. Throughout the heating process, the loss modulus (G″) was continuously recorded to monitor viscoelastic changes in the meat system [23].

### 2.12. Statistical Analysis

For each treatment group—control, 10% native whey protein (NWP), 5% Fraction I (FI), 10% FI, 15% FI, and 0.02% butylated hydroxyanisole (BHA)—the experiment comprised three independent biological replicates (*n* = 3), with each replicate derived from the longissimus dorsi muscle of a separate pig. All statistical analyses were performed using SAS software (version 9.2; SAS Institute Inc., Cary, NC, USA). Three independent biological replicates (*n* = 3) were used for all experimental treatments unless noted otherwise. To evaluate the effects of different treatments across varying freeze–thaw stages, the following analytical strategy was employed: For each measured parameter, at each fixed freeze–thaw cycle count (0, 1, 3, 5, 7 cycles), a one-way analysis of variance (ANOVA) was employed to compare differences between treatment groups. Where ANOVA indicated significant differences, Duncan’s multiple range test was used for post-hoc multiple comparisons at a significance level of *p* < 0.05. Experimental results are presented as mean ± standard deviation (SD).

## 3. Results and Discussion

### 3.1. DPPH Free Radical Scavenging Capacity

**Figure 1:**
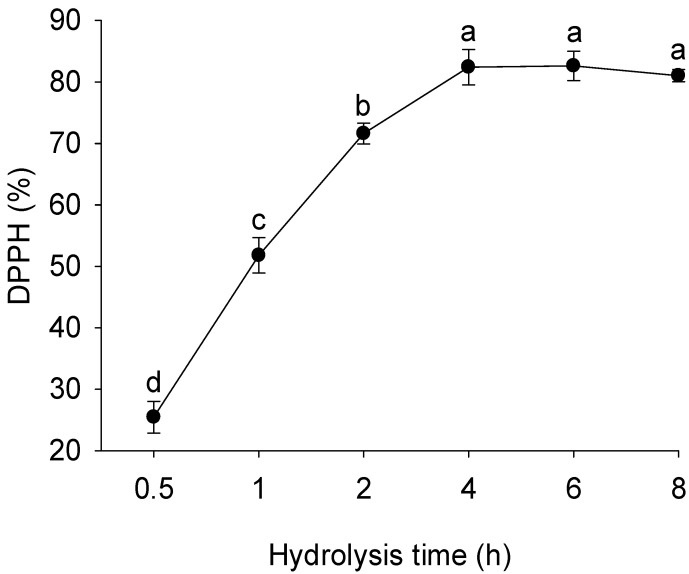
Variation in the DPPH radical scavenging capacity of whey protein hydrolysates prepared at different hydrolysis times. Values with different lowercase letters (a–d) differ significantly (*p* < 0.05); the same lettering system is used for all figures.

Because of its relative chemical stability, 2,2-diphenyl-1-picrylhydrazyl (DPPH) has been widely employed as a model radical for assessing the antioxidant activity of bioactive compounds [24]. DPPH is a stable nitrogen-centered free radical that readily reacts with antioxidant molecules capable of donating hydrogen atoms or electrons, resulting in a reduction in its characteristic violet coloration. As shown in Figure 1, the DPPH scavenging efficiency of whey protein hydrolysate (WPH) increased progressively with longer enzymatic hydrolysis, reaching the highest activity (82.6%) after 4 h of treatment. Beyond this duration, a slight, statistically insignificant decrease was observed (*p* > 0.05). These findings are consistent with those of Millan et al. [25], who reported that extended hydrolysis enhances the radical-scavenging potential of WPH up to approximately 4 h. The improved activity can be attributed to the increased exposure of peptide residues containing hydrogen or electron donors that stabilize free radicals through redox reactions, leading to the formation of more stable molecular structures [26]. Furthermore, prolonged enzymatic treatment may partially unfold the whey protein structure, producing smaller peptides or free amino acids with higher surface reactivity and stronger radical-quenching capacity [27]. Collectively, these results suggest that moderate hydrolysis conditions favor the formation of low-molecular-weight peptides with superior antioxidant potential, with optimal DPPH radical scavenging activity achieved at approximately 4 h of hydrolysis—representing a balance between peptide generation and the onset of degradation processes that could reduce antioxidant efficacy.

### 3.2. FRAP Assay

**Figure 2:**
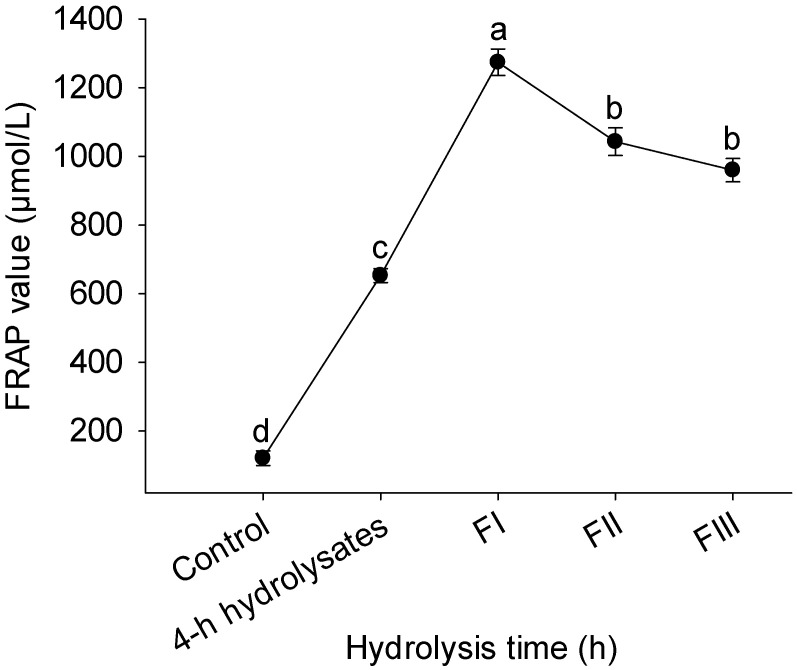
Effects of 4-hour hydrolysates and its different molecular weight components on the ferric-reducing antioxidant power (FRAP). FI, <1 kDa; FII, 1~3 kDa; FIII, >3 kDa. Values with different lowercase letters (a–d) differ significantly (*p* < 0.05).

The Ferric Reducing Antioxidant Power (FRAP) assay evaluates the ability of antioxidants to convert ferric ions (Fe^3+^) into their ferrous form (Fe^2+^), thereby providing an indication of a compound’s overall reducing capacity [28]. Figure 2 presents the FRAP activities of whey protein hydrolysates with different molecular weight ranges obtained after 4 h of enzymatic hydrolysis. All peptide fractions (FI, FII, and FIII) demonstrated significantly higher reducing power compared with both the unfractionated 4 h hydrolysate and the untreated whey protein (*p* < 0.05). Among them, FI—composed of peptides smaller than 1 kDa—displayed the strongest reducing potential, reaching a FRAP value of 1274.2 μmol/L, which was significantly higher than that of both FII and FIII (*p* < 0.05). Although the reducing ability followed a descending order of FI > FII > FIII, no significant difference was observed between the FRAP values of FII and FIII (*p* > 0.05). This indicates that the superior antioxidant capacity is primarily attributed to the lowest molecular weight fraction.

This pattern is in agreement with the findings of Kumar et al. [29], who reported that camel cheese protein hydrolysates within the 1–5 kDa range exhibited superior antioxidant and antibacterial activities. Similarly, Kong et al. [30] observed that the peptide fraction of whey protein isolate (0.1–2.8 kDa) showed the greatest antioxidant performance. However, the optimal molecular weight range for antioxidant activity can vary depending on the protein source and hydrolysis conditions, as demonstrated by Kumar et al. [29]. Vo et al. [31] further proposed that peptides of smaller size interact more efficiently with ferric ions due to their enhanced mobility and accessibility to redox sites, resulting in stronger ferric-reducing effects. The cleavage of peptide bonds during hydrolysis exposes functional side chains (e.g., thiol and carboxyl groups), which can donate protons and electrons to reduce Fe^3+^ ions, thereby enhancing the reducing capacity. Given that low-molecular-weight peptides appear to play a predominant role in the antioxidant mechanisms of whey protein hydrolysates, we selected Fraction I (FI)—which exhibited the highest FRAP value—for subsequent incorporation into ground pork to investigate its efficacy against oxidation during repeated freeze–thaw cycles.

### 3.3. Hydroxyl Radical Analysis

**Figure 3:**
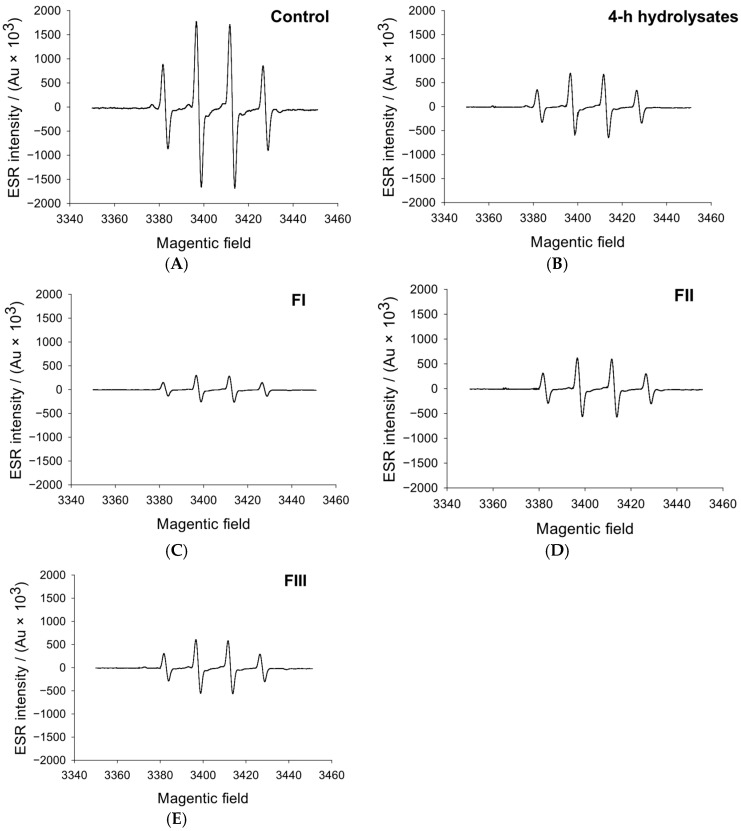
Effects of 4-h hydrolysate and its different molecular weight fractions on hydroxyl radicals. (**A**–**E**): Control, 4-h hydrolysates, FI, FII, FIII. FI, <1 kDa; FII, 1–3 kDa; FIII, >3 kDa.

Hydroxyl radicals (·OH) are among the most reactive oxygen species responsible for initiating lipid and protein oxidation in biological materials. Li et al. [32] reported that these radicals promote double-bond rearrangement in hydrogen peroxide and accelerate radical propagation reactions, thereby influencing the oxidative stability of meat systems and microbial structures. As shown in Figure 3, the hydroxyl radical scavenging activity of whey protein hydrolysate fractions varied markedly with molecular weight. The <1 kDa fraction (FI) exhibited the strongest scavenging performance, followed by the >3 kDa (FIII) and 1–3 kDa (FII) fractions. The FI fraction from the 4 h hydrolysate displayed approximately a 37% higher scavenging efficiency than the unfractionated sample (*p* < 0.05), whereas FII and FIII showed only modest increases of 7.1% and 8.3%, respectively. This inverse relationship between molecular weight and antioxidant activity [33] suggests that smaller peptides possess superior radical-neutralizing potential. The enhanced efficacy of FI may be attributed to a potentially higher proportion of hydrophobic amino acids, such as phenylalanine (F), alanine (A), proline (P), and glycine (G), which are known to enhance the interaction of peptides with reactive oxygen species. Wang et al. [34] emphasized that such hydrophobic residues facilitate peptide alignment at the lipid–water interface, enabling more efficient hydrogen or electron donation. It is plausible that the pronounced hydroxyl radical scavenging capacity of the <1 kDa fraction stems from the combined effect of this putative hydrophobic character and its smaller molecular size, which together enhance its ability to neutralize reactive radicals.

### 3.4. Superoxide Radical Analysis

**Figure 4:**
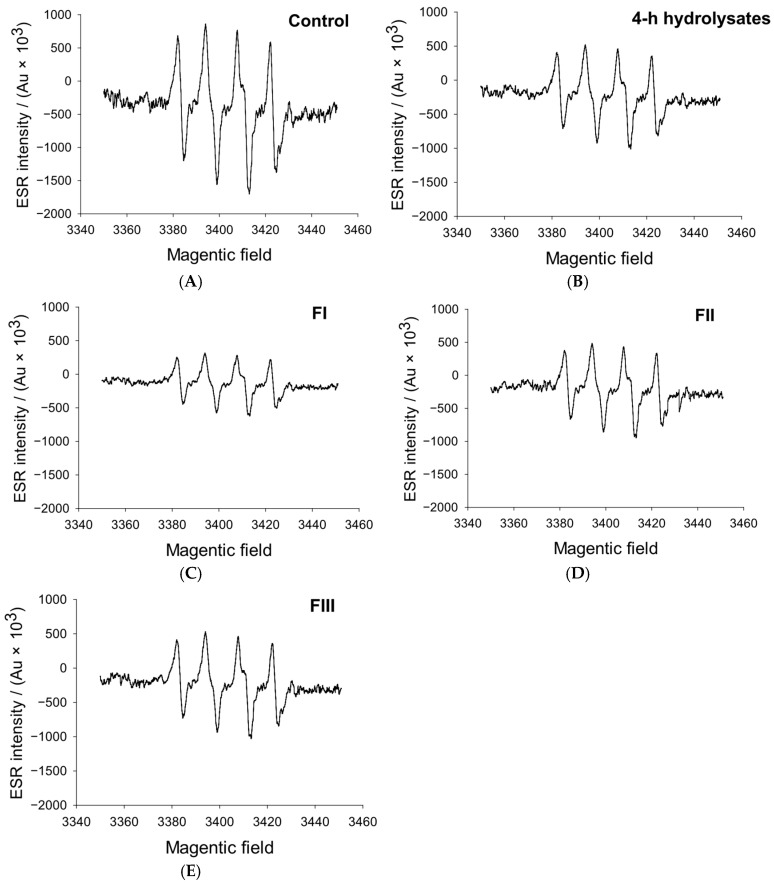
Effect of 4-hour hydrolysate and its different molecular weight fractions on superoxide radicals. (**A**–**E**): Control, 4-h hydrolysates, FI, FII, FIII. FI, <1 kDa; FII, 1–3 kDa; FIII, >3 kDa.

Superoxide radicals (O_2_•^−^) are highly reactive oxygen species that accelerate lipid oxidation and deteriorate meat quality during processing and storage [35]. As shown in Figure 4, distinct differences were observed in the scavenging efficiency of whey protein hydrolysate fractions with different molecular weights. The <1 kDa fraction (FI) exhibited the highest superoxide scavenging activity, achieving a 43% reduction rate that was significantly greater than that of the unfractionated 4 h hydrolysate (*p* < 0.05), indicating a greater accumulation of antioxidant-active components within this fraction. In contrast, the 1–3 kDa (FII) and >3 kDa (FIII) fractions displayed no notable differences in scavenging activity compared with the control (*p* > 0.05). The inverse relationship between peptide size and antioxidant activity, consistent with trends observed in the DPPH assay, suggests that smaller peptides possess stronger radical-neutralizing capacity [36]. This superior efficiency can be attributed to the higher content of bioactive peptides in FI, which contain multiple electron- and hydrogen-donating groups capable of stabilizing reactive oxygen species. Larger peptides, by contrast, likely include structurally rigid or partially degraded fragments with reduced redox potential, leading to weaker antioxidant effects [37]. Similarly, Karimi et al. [38] demonstrated that lower molecular weight peptides from hydrolyzed corn germ proteins exhibited markedly stronger antioxidant activity than their larger counterparts. Collectively, these findings confirm that the superoxide scavenging capability of whey protein hydrolysates is mainly concentrated in the <1 kDa fraction (FI), where small, highly reactive peptides provide enhanced oxidative protection through direct interaction and neutralization of reactive oxygen species.

### 3.5. DPPH Radical Analysis

**Figure 5:**
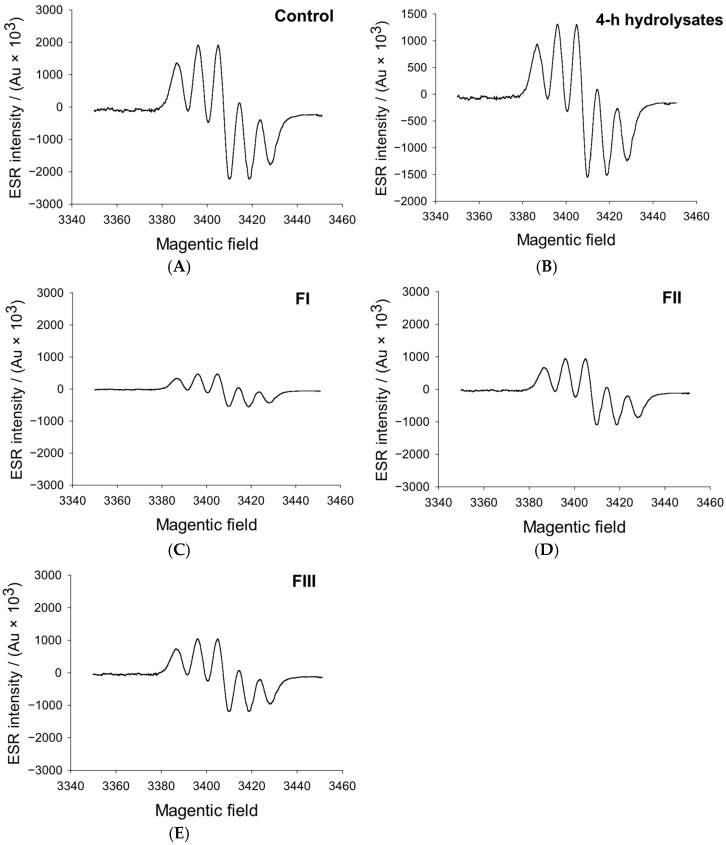
Effect of 4-hour hydrolysate and its different molecular weight fractions on DPPH radicals. (**A**–**E**): Control, 4-h hydrolysates, FI, FII, FIII. FI, <1 kDa; FII, 1–3 kDa; FIII, >3 kDa.

The DPPH radical scavenging assay is widely recognized as a simple and reliable approach for assessing the antioxidant potential of peptides and other bioactive substances derived from food proteins [39]. This method operates on the principle that antioxidants donate hydrogen atoms or electrons to the stable DPPH radical, reducing its characteristic violet coloration and consequently lowering absorbance. As shown in Figure 5, the whey protein hydrolysate fractions obtained by ultrafiltration after 4 h of enzymatic hydrolysis displayed marked differences in DPPH radical scavenging capacity. The fractions FI, FII, and FIII all demonstrated significantly greater activity than the unfractionated hydrolysate (*p* < 0.05). Among these, FI exhibited the strongest scavenging ability (75.85 ± 2.53ᵃ), surpassing both FII (51.24 ± 1.01ᵇ) and FIII (46.08 ± 2.37ᵇ). This pattern indicates that low-molecular-weight peptides (<1 kDa) have superior antioxidant efficacy. These results align with findings by Liu et al. [40], who observed that peptides derived from papain-hydrolyzed alligator meat exhibited similar size-dependent radical scavenging behavior. The enhanced antioxidant activity of FI may be linked to its enrichment in sulfur-containing amino acids such as methionine and cysteine, which act as key precursors in antioxidant biosynthesis and play an essential role in free-radical neutralization [41]. Moreover, the enzymatic hydrolysis of whey proteins likely generates new bioactive peptide sequences that contribute further to the radical-scavenging potential of this fraction [42]. Taken together, these results suggest that antioxidant activity in 4 h whey protein hydrolysate is mainly concentrated in the <1 kDa fraction, where small peptides with favorable physicochemical characteristics and reactive amino acid residues enhance the overall antioxidant performance. Consequently, incorporation of fi peptides into frozen-thawed ground pork systems may effectively suppress lipid oxidation through potent free-radical scavenging, thereby improving oxidative stability and preserving product quality during storage.

### 3.6. Acid Value

**Figure 6:**
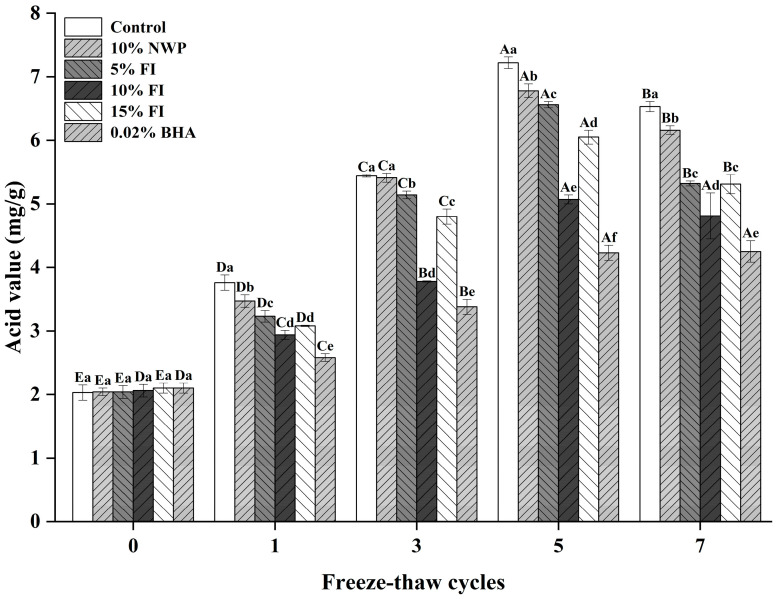
Acid value changes in ground pork with varying FI contents during freeze–thaw (F–T) cycles. Different uppercase letters (A–E) indicate significant differences among cycles within the same treatment; different lowercase letters (a–f) indicate significant differences among treatments within the same cycle. Control: without additives; NWP: native whey protein; BHA: butylated hydroxyanisole.

The acid value reflects the concentration of free fatty acids released during lipid hydrolysis and serves as an important indicator of fat deterioration in food systems [43]. As shown in Figure 6, all six treatment groups initially exhibited comparable acid values of approximately 2.05 mg/g prior to any freeze–thaw (F–T) cycles, with no significant differences detected (*p* > 0.05). However, as the number of F–T cycles increased, a gradual elevation in acid value was observed in all samples, reaching a peak after the fifth cycle. This trend may be associated with the reactivation of endogenous lipolytic enzymes following repeated exposure to subzero stress [43]. Moreover, successive freezing and thawing promote uneven ice crystal formation, disrupting muscle fiber structures and releasing lipase enzymes that accelerate lipid hydrolysis and rancidity [44]. Notably, the incorporation of Fraction I (FI) effectively slowed the increase in acid value, indicating partial inhibition of lipid hydrolysis. The greatest protective effect was achieved when FI was added at a 10% concentration, suggesting its potential to maintain the physicochemical stability of ground pork during repeated freeze–thaw cycles.

### 3.7. Peroxides Value

**Figure 7:**
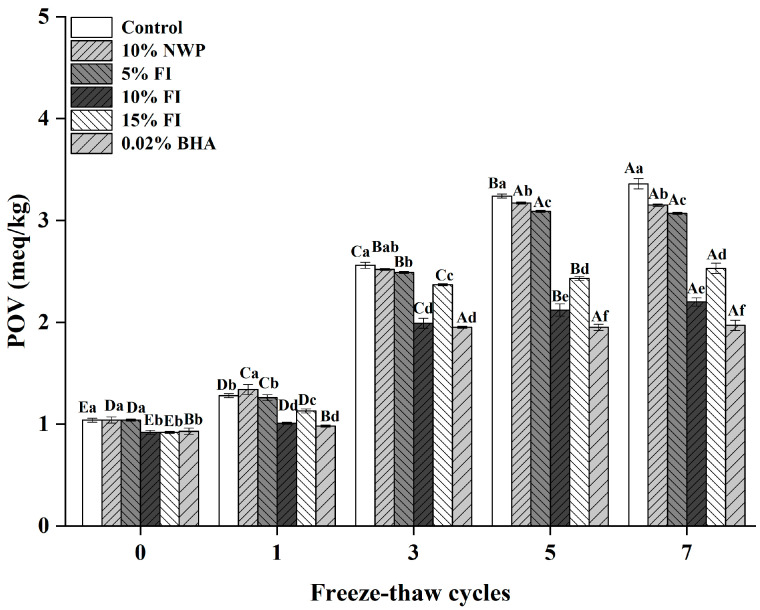
Peroxide value (POV) changes in ground pork with varying FI contents over multiple freeze–thaw (F–T) cycles. Different uppercase letters (A–E) indicate significant differences among cycles within the same treatment; different lowercase letters (a–f) indicate significant differences among treatments within the same cycle. Control: without additives; NWP: native whey protein; FI: Fraction I of whey protein hydrolysates; BHA: butylated hydroxyanisole.

The peroxide value (POV) serves as a critical indicator of primary lipid oxidation in meat products, reflecting the content of hydroperoxides formed during the initial stages of oxidation [45]. As illustrated in Figure 7, the initial POV of ground pork prior to freeze–thaw (F–T) cycles ranged between 0.92 and 1.04 meq/kg across all groups. With successive F–T cycles, the POV gradually increased in all samples, reaching a maximum of 1.97–3.36 meq/kg after seven cycles, with statistically significant differences observed among the treatment groups (*p* < 0.05). This trend is consistent with previous findings [13] and can be attributed to the release and activation of endogenous pro-oxidants (e.g., heme iron) and the disruption of muscle structure induced by ice crystal formation during repeated freezing and thawing, which collectively promote lipid oxidation [46].

After seven F–T cycles, the increases in POV relative to the initial values were 2.09, 2.03, 1.27, 1.61, and 1.04 meq/kg for samples supplemented with 10% NWP, 5% FI, 10% FI, 15% FI, and 0.02% BHA, respectively. All of these values were significantly lower than those of the untreated control (*p* < 0.05). Notably, the 10% FI and 0.02% BHA groups exhibited the lowest POV levels throughout the F–T cycles, indicating a strong antioxidant capacity comparable to that of the synthetic antioxidant BHA. These results suggest that 10% FI effectively scavenges lipid radicals and suppresses the propagation of lipid oxidation. However, the fact that 15% FI did not confer additional protection suggests a possible plateau or solubility-limited effect at higher concentrations.

### 3.8. TBARS

**Figure 8:**
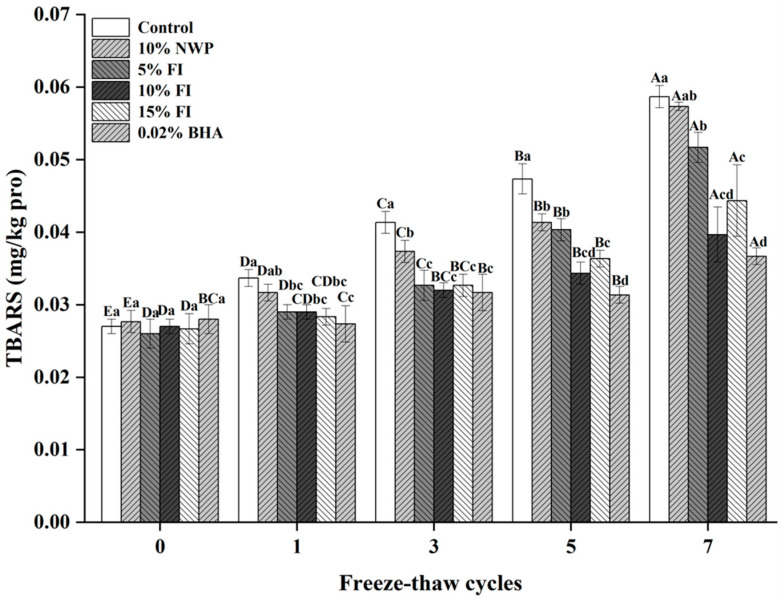
TBARS value changes in ground pork with varying FI contents over multiple freeze–thaw (F–T) cycles. Different uppercase letters (A–E) indicate significant differences among cycles within the same treatment; different lowercase letters (a–d) indicate significant differences among treatments within the same cycle. Control: without additives; NWP: native whey protein; FI: Fraction I of whey protein hydrolysates; BHA: butylated hydroxyanisole.

The thiobarbituric acid reactive substances (TBARS) value serves as an important index for assessing lipid oxidation, as it quantifies malondialdehyde (MDA)—a major secondary oxidation product formed during lipid peroxidation. Elevated MDA levels reflect greater oxidative deterioration of lipids [47]. As shown in Figure 8, TBARS values for all six treatment groups were initially around 0.027 mg/kg at 0 freeze–thaw (F–T) cycles, with no significant differences among groups (*p* > 0.05). With successive F–T cycles, the TBARS values gradually increased, showing a pronounced rise after the third cycle and peaking after the seventh, which aligns with the observations of Pan et al. [13]. This acceleration can be attributed to the release of pro-oxidant molecules following muscle microstructure disruption caused by ice crystal formation during repeated freezing and thawing [5]. Additionally, the F–T process promotes the leakage of endogenous enzymes such as lipases, proteases, and oxidases, further catalyzing oxidative reactions [48]. Consequently, repeated F–T cycles, compared with storage of fresh pork, markedly intensify lipid oxidation and lead to muscle tissue degradation and protein denaturation. Consistent with previous reports, the inclusion of antioxidants effectively reduced TBARS levels, thereby mitigating oxidative damage and preserving product quality [49]. After seven cycles, the untreated control displayed the highest TBARS value (0.059 mg/kg), while the group containing 0.02% butylated hydroxyanisole (BHA) had the lowest (0.037 mg/kg), showing a statistically significant difference (*p* < 0.05). Remarkably, the sample supplemented with 10% Fraction I (FI) showed a TBARS value of 0.04 mg/kg, comparable to that of the BHA-treated group. These findings highlight that FI exhibits antioxidant efficacy similar to that of synthetic antioxidants and may serve as a natural alternative for inhibiting lipid rancidity and enhancing the oxidative stability of ground pork products.

### 3.9. pH

**Figure 9:**
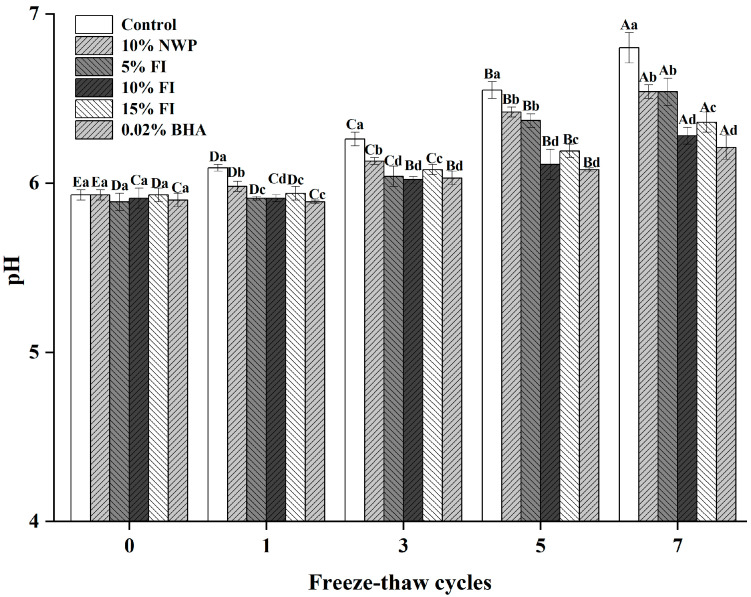
pH changes in ground pork with varying FI contents over multiple freeze–thaw (F–T) cycles. Different uppercase letters (A–E) indicate significant differences among cycles within the same treatment; different lowercase letters (a–d) indicate significant differences among treatments within the same cycle. Control: without additives; NWP: native whey protein; FI: Fraction I of whey protein hydrolysates; BHA: butylated hydroxyanisole.

Meat pH serves as a critical marker for its freshness and stability, as it exerts a significant impact on key quality characteristics like color, water-holding capacity, and overall texture [22]. Figure 9 demonstrates that the initial pH levels of all ground pork groups, measured prior to freeze–thaw cycles, ranged from 5.89 to 5.93, and no significant differences were detected among these groups (*p* > 0.05). Nevertheless, with the increase in the number of freeze–thaw (F–T) cycles, a significant rise in pH was detected in the control group (*p* < 0.05). This pH elevation is likely related to the microbial decomposition and enzymatic breakdown of proteins and other nitrogenous compounds during storage, which can generate alkaline metabolites such as ammonia and amines that gradually elevate pH levels [33]. After three F–T cycles, statistically significant differences appeared between the control and treated samples (*p* < 0.05). Notably, pork treated with 10% Fraction I (FI) or 0.02% butylated hydroxyanisole (BHA) maintained significantly lower pH values compared to untreated samples, indicating a slower rate of protein and lipid degradation. These results demonstrate that supplementation with 10% FI can effectively mitigate the pH increase associated with repeated freeze–thaw cycles, showing antioxidant and preservative efficacy comparable to that of 0.02% BHA.

### 3.10. G″ Value

**Figure 10:**
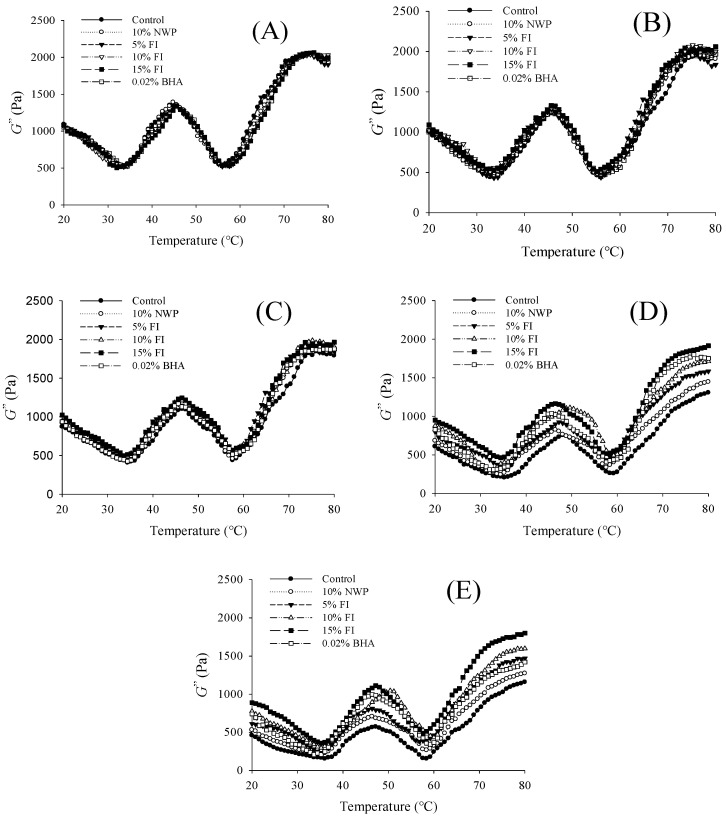
Rheological profiles of ground pork with different FI contents over 0–7 freeze–thaw (F–T) cycles. (**A**–**E**): 0, 1, 3, 5, and 7 cycles.

The G″ value represents the viscous response of the ground pork gel matrix and reflects the amount of mechanical energy dissipated as heat during deformation, thus characterizing the viscous component of the system [50]. As shown in Figure 10, all samples exhibited a typical rheological pattern across successive freeze–thaw (F–T) cycles. During heating, the G″ value initially declined with increasing temperature and then rose steadily, reaching its first peak around 47 °C. A second rise occurred after approximately 58 °C, with a maximum near 78 °C, followed by a slight decrease. The first peak likely corresponds to myosin head aggregation, exposure of hydrophobic domains, and the melting of intramuscular fat [51]. The subsequent reduction after the second peak may result from the formation of a dense, irreversible gel network produced by protein cross-linking and thermal denaturation. After seven F–T cycles, all groups displayed a notable decrease in G″ values, indicating structural weakening of the gel system. Nevertheless, the samples containing natural whey protein (NWP), Fraction I (FI), or butylated hydroxyanisole (BHA) maintained higher G″ values than the untreated control, suggesting improved viscoelastic stability. Among the FI treatment groups, the 15% FI group exhibited the maximum G″ value, with 10% FI ranking second and only slight discrepancies between the two. These results indicate that the incorporation of FI peptides can effectively alleviate the reduction in viscoelasticity of ground pork gels during repeated freeze–thaw cycles. The enhanced rheological properties may be ascribed to the antioxidant activity of FI, which prevents proteins from undergoing oxidative cross-linking, promotes the exposure of hydrophobic sites, and boosts the formation of stable gel networks, thereby ultimately enhancing both the texture and water-holding capacity [52].

## 4. Conclusions

In conclusion, this study demonstrated that the low-molecular-weight fraction of whey protein hydrolysate (Fraction I, <1 kDa) exhibited strong antioxidant activity, effectively scavenging DPPH, superoxide, and hydroxyl radicals in vitro. When incorporated into ground pork, FI significantly suppressed the increase in oxidative indicators (peroxide value, TBARS), chemical spoilage markers (acid value, pH), and the loss of viscoelasticity (G″ value) associated with repeated freeze–thaw (F–T) cycles. Among all treatments, 10% FI achieved the most pronounced inhibition of lipid oxidation, performing nearly as well as 0.02% butylated hydroxyanisole (BHA), while 15% FI most effectively preserved the gel’s viscous property (G″ value), likely due to enhanced water-binding and network stability. Overall, supplementation with FI peptides offers a natural and effective approach for improving oxidative stability, mitigating chemical spoilage, and enhancing the rheological properties of ground pork subjected to multiple freeze–thaw cycles. Despite the promising results, this study has certain limitations. The relatively high incorporation level of FI (10–15%) and the use of a model meat system necessitate further investigation into its cost-effectiveness, sensory impact, and efficacy in complex food matrices at an industrial scale. Future work will focus on optimizing the dosage, evaluating its effects on sensory attributes, and validating its application in commercial meat products.

## Data Availability

The original contributions presented in the study are included in the article, further inquiries can be directed to the corresponding author.

## References

[1] Zhang R., Realini C.E., Kim Y.H.B., Farouk M.M. (2023). Challenges and processing strategies to produce high quality frozen meat. Meat Sci..

[2] Xie A., Sun D.-W., Xu Z., Zhu Z. (2015). Rapid detection of frozen pork quality without thawing by Vis–NIR hyperspectral imaging technique. Talanta.

[3] Zhang M., Li F., Diao X., Kong B., Xia X. (2017). Moisture migration, microstructure damage and protein structure changes in porcine longissimus muscle as influenced by multiple freeze-thaw cycles. Meat Sci..

[4] Kong C.H.Z., Hamid N., Ma Q., Lu J., Wang B.-G., Sarojini V. (2017). Antifreeze peptide pretreatment minimizes freeze-thaw damage to cherries: An in-depth investigation. LWT.

[5] Li F., Zhong Q., Kong B., Wang B., Pan N., Xia X. (2020). Deterioration in quality of quick-frozen pork patties induced by changes in protein structure and lipid and protein oxidation during frozen storage. Food Res. Int..

[6] Pan N., Dong C., Du X., Kong B., Sun J., Xia X. (2021). Effect of freeze-thaw cycles on the quality of quick-frozen pork patty with different fat content by consumer assessment and instrument-based detection. Meat Sci..

[7] Wen R., Hu Y., Zhang L., Wang Y., Chen Q., Kong B. (2019). Effect of NaCl substitutes on lipid and protein oxidation and flavor development of Harbin dry sausage. Meat Sci..

[8] Qi J., Li C., Chen Y., Gao F., Xu X., Zhou G. (2012). Changes in meat quality of ovine longissimus dorsi muscle in response to repeated freeze and thaw. Meat Sci..

[9] Lorido L., Ventanas S., Akcan T., Estévez M. (2016). Effect of protein oxidation on the impaired quality of dry-cured loins produced from frozen pork meat. Food Chem..

[10] Wang B., Li F., Pan N., Kong B., Xia X. (2021). Effect ofice structuring protein on the quality of quick-frozen patties subjected to multiple freeze-thaw cycles. Meat Sci..

[11] Lin S., Yang S., Li X., Chen F., Zhang M. (2016). Dynamics of water mobility and distribution in soybean antioxidant peptide powders monitored by LF-NMR. Food Chem..

[12] Zhao C., Gong Y., Zheng L., Zhao M. (2023). Whey protein hydrolysate enhances exercise endurance, regulates energy metabolism, and attenuates muscle damage in exercise mice. Food Biosci..

[13] Ha H.-K., Rankin S., Lee M.-R., Lee W.-J. (2019). Development and Characterization of Whey Protein-Based Nano-Delivery Systems: A Review. Molecules.

[14] Li Y., Kong B., Xia X., Liu Q., Diao X. (2013). Structural changes of the myofibrillar proteins in common carp (*Cyprinus carpio*) muscle exposed to a hydroxyl radical-generating system. Process Biochem..

[15] Liu C., Kong L., Yu P., Wen R., Yu X., Xu X., Peng X. (2022). Whey Protein Hydrolysates Improved the Oxidative Stability and Water-Holding Capacity of Pork Patties by Reducing Protein Aggregation during Repeated Freeze–Thaw Cycles. Foods.

[16] Peng X., Xiong Y.L., Kong B. (2009). Antioxidant activity of peptide fractions from whey protein hydrolysates as measured by electron spin resonance. Food Chem..

[17] Shen D.-Y., Begum N., Song H.-L., Zhang Y., Wang L.-J., Zhao Y.-J., Zhang L., Liu P. (2021). In vitro and in vivo antioxidant activity and umami taste of peptides (<1 kDa) from porcine bone protein extract. Food Biosci..

[18] Chen Y., Huang F., Xie B., Sun Z., McClements D.J., Deng Q. (2021). Fabrication and characterization of whey protein isolates- lotus seedpod proanthocyanin conjugate: Its potential application in oxidizable emulsions. Food Chem..

[19] Liu W., Tang D., Ao C. (2022). Adding of Allium mongolicum regel extracts to lamb feedlot diets influences 4-alkyl-branched fatty acids deposition and the meat quality during storage. Meat Sci..

[20] Wang Z., He Z., Gan X., Li H. (2018). Interrelationship among ferrous myoglobin, lipid and protein oxidations in rabbit meat during refrigerated and superchilled storage. Meat Sci..

[21] Gan S., Zhang M., Mujumdar A.S., Jiang Q. (2022). Effects of different thawing methods on quality of unfrozen meats. Int. J. Refrig..

[22] Hu R., Zhang M., Mujumdar A.S. (2022). Application ofinfrared and microwave heating prior to freezing of pork: Effect on frozen meat quality. Meat Sci..

[23] Zhao X., Bai Y., Xing T., Xu X.-l., Zhou G. (2018). Use of an isoelectric solubilization/precipitation process to modify the functional properties of PSE (pale, soft, exudative)-like chicken meat protein: A mechanistic approach. Food Chem..

[24] Jang H.L., Liceaga A.M., Yoon K.Y. (2016). Purification, characterisation and stability of an antioxidant peptide derived from sandfish (Arctoscopus japonicus) protein hydrolysates. J. Funct. Foods.

[25] Millan G.C.L., Veras F.F., Stincone P., Pailliè-Jiménez M.E., Brandelli A. (2022). Biological activities of whey protein hydrolysate produced by protease from the Antarctic bacterium Lysobacter sp. A03. Biocatal. Agric. Biotechnol..

[26] Gomes M.H.G., Kurozawa L.E. (2020). Improvement of the functional and antioxidant properties of rice protein by enzymatic hydrolysis for the microencapsulation of linseed oil. J. Food Eng..

[27] Phongthai S., Lim S.-T., Rawdkuen S. (2016). Optimization of microwave-assisted extraction of rice bran protein and its hydrolysates properties. J. Cereal Sci..

[28] Tkaczewska J., Borawska-Dziadkiewicz J., Kulawik P., Duda I., Morawska M., Mickowska B. (2020). The effects of hydrolysis condition on the antioxidant activity of protein hydrolysate from Cyprinus carpio skin gelatin. LWT.

[29] Kumar D., Chatli M.K., Singh R., Mehta N., Kumar P. (2016). Antioxidant and antimicrobial activity of camel milk casein hydrolysates and its fractions. Small Rumin. Res..

[30] Kong B., Peng X., Xiong Y.L., Zhao X. (2012). Protection of lung fibroblast MRC-5 cells against hydrogen peroxide-induced oxidative damage by 0.1–2.8kDa antioxidative peptides isolated from whey protein hydrolysate. Food Chem..

[31] Vo H., Saldaña M.D.A. (2023). Hydrolysis of pea protein concentrate in subcritical water media with addition of citrus pectin and citric acid. J. Supercrit. Fluids.

[32] Li S., Tang S., Li J., Chen L., Ma Y. (2022). Protective Effects of Four Natural Antioxidants on Hydroxyl-Radical-Induced Lipid and Protein Oxidation in Yak Meat. Foods.

[33] Kong L., Liu C., Tang H., Yu P., Wen R., Peng X., Xu X., Yu X. (2023). Hygroscopicity and antioxidant activity of whey protein hydrolysate and its ability to improve the water holding capacity of pork patties during freeze−thaw cycles. LWT.

[34] Wang L., Li Z., Fan X., Zhang T., Wang H., Ye K. (2024). Novel antioxidant peptides from bovine blood: Purification, identification and mechanism of action. LWT.

[35] Zhang X., Guo L., Hong C., Wu P., Tuly J., Ma H. (2025). Accumulation of phenolic in fresh-cut lotus roots induced by thermosonication: Regulation of phenylpropanoid pathway and reactive oxygen species metabolism. Food Chem..

[36] Unuofin J.O., Oladipo A.O., More G.K., Adeeyo A.O., Mustapha H.T., Msagati T.A.M., Lebelo S.L. (2024). Phytochemical Profiling of Phragmites australis Leaf Extract and Its Nano-Structural Antioxidant, Antimicrobial, and Anticancer Activities. J. Ofinorganic Organomet. Polym. Mater..

[37] Manessis G., Kalogianni A.I., Lazou T., Moschovas M., Bossis I., Gelasakis A.I. (2020). Plant-Derived Natural Antioxidants in Meat and Meat Products. Antioxidants.

[38] Karimi A., Azizi M.H., Ahmadi Gavlighi H. (2020). Fractionation of hydrolysate from corn germ protein by ultrafiltration: In vitro antidiabetic and antioxidant activity. Food Sci. Nutr..

[39] Yang D., Zhang L., Gao S., Luo Y., Luo R., Hou Y. (2025). Effects of grape seed proanthocyanidin on the conformation and functional properties of lamb myofibrillar protein under hydroxyl radical-induced oxidative stress. LWT.

[40] Yang L., Xing Y., Chen R., Ni H., Li H.-H. (2022). Isolation and identification of antioxidative peptides from crocodile meat hydrolysates using silica gel chromatography. Sci. Rep..

[41] Ramani A., Hazra T., Mudgil S., Mudgil D. (2024). Emerging potential of whey proteins in prevention of cancer. Food Humanit..

[42] Cui L., Yang G., Lu S., Zeng X., He J., Guo Y., Pan D., Wu Z. (2022). Antioxidant peptides derived from hydrolyzed milk proteins by Lactobacillus strains: A BIOPEP-UWM database-based analysis. Food Res. Int..

[43] Otoo E.A., Ocloo F.C.K., Appiah V. (2022). Effect of gamma irradiation on shelf life of smoked guinea fowl (*Numida meleagris*) meat stored at refrigeration temperature. Radiat. Phys. Chem..

[44] Jiang Q., Nakazawa N., Hu Y., Osako K., Okazaki E. (2019). Changes in quality properties and tissue histology of lightly salted tuna meat subjected to multiple freeze-thaw cycles. Food Chem..

[45] Rahman M.H., Alam M.S., Monir M.M., Ahmed K. (2021). Comprehensive effects of black cumin (*Nigella sativa*) and synthetic antioxidant on sensory and physicochemical quality of beef patties during refrigerant storage. J. Agric. Food Res..

[46] Al-Dalali S., Li C., Xu B. (2022). Effect of frozen storage on the lipid oxidation, protein oxidation, and flavor profile of marinated raw beef meat. Food Chem..

[47] Xia X., Kong B., Liu J., Diao X., Liu Q. (2012). Influence of different thawing methods on physicochemical changes and protein oxidation of porcine longissimus muscle. LWT—Food Sci. Technol..

[48] Sun Q., Chen Q., Xia X., Kong B., Diao X. (2019). Effects of ultrasound-assisted freezing at different power levels on the structure and thermal stability of common carp (*Cyprinus carpio*) proteins. Ultrason. Sonochemistry.

[49] Ahmad Mir S., Ahmad Masoodi F., Raja J. (2017). Influence of natural antioxidants on microbial load, lipid oxidation and sensorial quality of rista—A traditional meat product ofindia. Food Biosci..

[50] Chen X., Li X., Yang F., Wu J., Huang D., Huang J., Wang S. (2022). Effects and mechanism of antifreeze peptides from silver carp scales on the freeze-thaw stability of frozen surimi. Food Chem..

[51] Li M., He S., Sun Y., Pan D., Zhou C., He J. (2023). Effectiveness of l-arginine/l-lysine in retarding deterioration of structural and gelling properties of duck meat myofibrillar protein during freeze-thaw cycles. Food Biosci..

[52] Peng X., Liu C., Wang B., Kong L., Wen R., Zhang H., Yu X., Bai Y., Jang A. (2023). Hygroscopic properties of whey protein hydrolysates and their effects on water retention in pork patties during repeated freeze–thaw cycles. LWT.

